# Exosome-Associated Proteins as Mediators and Biomarkers of Ovarian Cancer Dissemination

**DOI:** 10.3390/biom16081150

**Published:** 2026-08-07

**Authors:** Aleksei Shefer, Ekaterina Ivanova, Alyona Chernyshova, Svetlana Tamkovich

**Affiliations:** Institute of Oncology and Neurosurgery, E.N. Meshalkin National Medical Research Center, Ministry of Health of the Russian Federation, 630055 Novosibirsk, Russia; a.shefer@g.nsu.ru (A.S.); e.dzhugashvili@g.nsu.ru (E.I.); chernyshova_a@meshalkin.ru (A.C.)

**Keywords:** ovarian cancer, exosomes, extracellular vesicles, exosomal proteome, peritoneal dissemination, liquid biopsy, protein biomarkers, proteomics

## Abstract

Ovarian cancer (OC) remains the most lethal gynecological malignancy, mostly due to its frequent diagnosis at advanced stages, early peritoneal dissemination, ascites formation, and limited sensitivity of currently available approaches for early detection. Extracellular vesicles (EVs), particularly exosomes, mediate intercellular communication through the transfer of proteins, lipids, metabolites, and nucleic acids. In OC, EV-associated protein profiles reflect both tumor-cell-intrinsic programs and the complex interactions between malignant cells and the peritoneal microenvironment. This review summarizes current evidence regarding the involvement of exosomal proteins in OC progression, with particular emphasis on epithelial–mesenchymal transition, mesothelial reprogramming, extracellular matrix remodeling, angiogenesis, immune suppression, peritoneal dissemination, and platinum resistance. Mechanistic studies indicate that exosomal proteins, including CD44, the integrin α5β1/asparaginyl endopeptidase complex, annexin A2, low-density lipoprotein receptor-related protein 1, and programmed death-ligand 1, can directly contribute to metastatic niche formation and tumor progression. In parallel, proteomic studies of plasma-, serum-, ascites-, peritoneal-fluid-, and uterine-lavage-derived EVs have identified candidate liquid-biopsy biomarkers, including MUC1, EpCAM, FOLR1, integrins, complement- and coagulation-related proteins, and proteins associated with treatment resistance. To integrate the biological significance of proteins reported in OC-associated exosomes, we additionally performed protein–protein interaction and functional enrichment analyses. These analyses revealed interconnected protein groups associated with cell adhesion, oxidative stress adaptation, secretory remodeling, lipid metabolism, extracellular matrix organization, and inflammatory signaling. Taken together, the available evidence supports exosomal proteome profiling as a promising approach for investigating OC dissemination and developing minimally invasive diagnostic and prognostic tools. However, standardized EV isolation, quantitative proteomics, functional validation, and independent clinical cohorts remain essential for translation into clinical practice.

## 1. Introduction

Ovarian cancer (OC) remains the most lethal gynecological malignancy and is characterized by early peritoneal dissemination, frequent ascites formation, and late clinical detection [1]. According to global cancer statistics, more than 300,000 new OC cases and over 200,000 deaths are registered annually worldwide, reflecting the high mortality-to-incidence ratio of this disease [2]. The poor prognosis is largely explained by the absence of specific early symptoms and the limited diagnostic performance of currently available tools. Only a minority of patients are diagnosed at International Federation of Gynecology and Obstetrics (FIGO) stages I–II, whereas most cases are detected after tumor spread within the peritoneal cavity [3,4,5]. Ovarian neoplasms are a heterogeneous group of tumors that includes epithelial, germ-cell, and sex cord–stromal neoplasms. Epithelial OC accounts for the vast majority of malignant cases, with high-grade serous ovarian carcinoma (HGSOC) being the dominant and most aggressive histotype [3,4]. In modern clinical practice, ovarian, fallopian tube, and primary peritoneal carcinomas are often considered together because of their overlapping biology, staging, and treatment principles, especially within the high-grade serous carcinoma spectrum [3,4]. This biological heterogeneity complicates early diagnosis and requires biomarkers that reflect not only tumor presence but also tumor origin, dissemination potential, and interaction with the microenvironment.

Current OC diagnostics rely on a combination of clinical assessment, transvaginal ultrasound, computed tomography (CT), MRI, serum biomarkers, and histological verification [4,5,6,7,8]. Although imaging modalities is indispensable for detecting adnexal masses, assessing tumor spread, and planning cytoreductive surgery, these imaging modalities have important limitations in early-stage disease. Transvaginal ultrasound is widely used as a first-line imaging modality and can provide high diagnostic accuracy when performed by expert operators, but its performance is strongly operator-dependent and decreases when lesions are small, morphologically ambiguous, or not clearly distinguishable from benign adnexal pathology [9]. MRI is the standard method for staging advanced OC and assessing resectability, yet it has limited sensitivity for small-volume peritoneal implants, superficial serosa lesions, and early microscopic dissemination [4,6]. MRI offers better soft-tissue contrast and is useful for indeterminate adnexal masses and pelvic disease characterization, but it is more expensive, less available, and still cannot reliably detect all early or microscopic tumor deposits [5,6]. Positron emission tomography/computed tomography (PET/CT) may improve the detection of metabolically active metastatic or recurrent disease, but its sensitivity for small primary lesions is limited, and it is not recommended as a routine screening or first-line diagnostic tool [5,6,7]. In ultrasound-based triage, standardized morphological models may partially improve the interpretation of adnexal masses. The International Ovarian Tumor Analysis (IOTA) approach distinguishes benign and malignant lesions using features such as unilocular cystic morphology, acoustic shadows and absent Doppler flow for benign masses, and irregular solid components, ascites, multiple papillary projections and high vascularization for malignant lesions [10]. In addition, the IOTA Assessment of Different NEoplasias in the adneXa (ADNEX) model combines clinical variables with ultrasound features to estimate the probabilities of a benign tumor, a borderline tumor, stage I invasive cancer, stage II–IV invasive cancer, or a secondary metastatic tumor [11]. However, these approaches improve risk stratification rather than overcome the fundamental limitations of ultrasound as a population screening tool. Cross-sectional imaging should therefore be considered mainly as a staging and treatment-planning instrument. CT and MRI allow evaluation of tumor size, pelvic invasion, lymph node involvement and relationships with adjacent organs, which is essential for estimating the feasibility of optimal cytoreduction. Radiological resectability criteria include involvement of the stomach, pancreas, duodenum, bowel and major vascular structures, such as the celiac trunk and hepatic artery [12]. Nevertheless, CT has important limitations: its sensitivity and specificity for defining optimal treatment strategy were reported as 79% and 75%, respectively, whereas performance for detecting metastatic lymph-node involvement was limited, with sensitivity and specificity of 41% and 89% [12]. Therefore, small peritoneal implants and the peritoneal carcinomatosis index should not be assessed using CT alone [12,13]. PET/CT may be useful as a problem-solving modality for suspected nodal or distant metastatic disease and for post-treatment assessment, but it should not be presented as a routine screening or first-line diagnostic method. Its value in primary diagnosis is limited by poor discrimination between benign and malignant adnexal masses, low fluorodeoxyglucose uptake in mucinous and clear-cell carcinomas, and physiological uptake in corpus luteum cysts in women of reproductive age [12]. Thus, conventional imaging modalities are most effective when a morphologically visible tumor mass or macroscopic dissemination is already present. This represents a fundamental limitation for early detection: imaging identifies structural consequences of tumor growth, but not necessarily the molecular changes that precede clinically detectable disease.

CA-125 remains the most widely used serum marker, but its diagnostic value is limited: it is not elevated in a substantial proportion of early-stage cases and may increase in benign inflammatory or gynecological conditions [14]. Human epididymis protein 4 (HE4) and combined algorithms, such as the Risk of Ovarian Malignancy Algorithm (ROMA), improve diagnostic specificity in selected settings, but they still do not provide sufficient accuracy for population screening or reliable early detection [7,15]. Large screening trials using CA-125-based algorithms and transvaginal ultrasound have not demonstrated a sufficient reduction in mortality to support routine screening of average-risk women [16]. Therefore, new minimally invasive approaches are urgently needed.

Liquid biopsy has emerged as a promising strategy for overcoming some limitations of tissue biopsy and conventional serum biomarkers. It enables analysis of tumor-derived and host-response molecules in accessible biological fluids, including blood plasma, serum, ascites, urine, and uterine lavage [17]. Among liquid biopsy analytes, extracellular vesicles (EVs), particularly small extracellular vesicles (sEVs) often referred to as exosomes, are of special interest. Exosomes are 30–150 nm lipid bilayer vesicles, carrying specific tetraspanins CD9, CD81 and CD63, secreted by all cell types and carrying proteins, lipids, metabolites, DNA, mRNA, microRNA, long noncoding RNAs and circular RNAs [18,19]. Their cargo reflects the physiological or pathological state of donor cells and can influence recipient cells through intercellular communication [17,19]. In OC, exosomes are not only passive carriers of molecular information, but also active participants in tumor progression [5,20]. They contribute to epithelial–mesenchymal transition (EMT), angiogenesis, vasculogenic mimicry, immune modulation, extracellular matrix remodeling, pre-metastatic niche formation, and peritoneal dissemination [5,21]. Exosomes are abundant in plasma and ascitic fluid of OC patients, and ascites-derived vesicles are particularly relevant because ascitic fluid represents a unique tumor microenvironment where cancer cells, immune cells, fibroblasts, mesothelial cells, and soluble mediators continuously interact [20]. Thus, exosomal cargo may reflect both tumor-cell-intrinsic programs and stromal or immune components of disease progression. Unlike our previous narrative review, which broadly addressed exosomal cargo, including proteins, lipids, and regulatory RNAs, in ovarian cancer dissemination [5], the present review focuses specifically on EV-associated proteins. It incorporates recent proteomic, targeted-proteomic, and single-EV studies and adds an integrative analysis of a curated protein set using protein–protein association networks, clustering, functional enrichment, and annotation-overlap analysis. Thus, the principal novelty of the present work lies in the protein-focused synthesis and the bioinformatic organization of the rapidly expanding OC EV proteomics literature.

## 2. Exosomal Proteins in OC Dissemination

OC dissemination is closely associated with the specific biology of this malignancy. Unlike many solid tumors, OC frequently spreads by exfoliation of malignant cells into the peritoneal cavity, followed by survival in ascitic fluid, spheroid formation, adhesion to the mesothelium, invasion of the submesothelial matrix, and growth of peritoneal implants. In this process, exosomes and other small EVs should not be considered merely by-products of tumor-cell activity. They represent biologically active mediators of intercellular communication between tumor cells, mesothelial cells, fibroblasts, macrophages, endothelial cells, and immune-cell populations within ascites and the peritoneal microenvironment [5,22].

EV-associated proteins may occupy different topological compartments, including the EV lumen, the lipid bilayer, and the external vesicle surface. This distinction is biologically important because surface-accessible proteins may interact directly with receptors, extracellular matrix components, or antibodies used for EV capture, whereas luminal proteins generally become accessible only after vesicle internalization, membrane fusion, or intracellular processing. MISEV2023 therefore recommends determining the localization of putatively active EV components using approaches such as limited proteolysis, membrane permeabilization, or affinity-reagent accessibility assays [18]. Integral membrane and surface-accessible proteins, including tetraspanins, integrins, CD44, PD-L1, and membrane-associated proteases, may participate in EV adhesion, recipient-cell recognition, immunocapture, and uptake [18,23]. By contrast, cytosolic, metabolic, and vesicle-trafficking proteins detected in EV preparations may be luminal, peripherally associated with the membrane, or co-isolated with vesicles, unless their topology has been experimentally established [18]. EVs circulating in biological fluids may additionally acquire a protein corona composed of extracellular proteins adsorbed onto their surface. Experimental incubation of EVs in human plasma demonstrated the association of apolipoproteins, complement components, fibrinogen, and immunoglobulins with the EV surface, indicating that at least some proteins commonly detected in plasma EV preparations may represent corona components rather than proteins loaded into EVs by donor cells [24]. This issue is particularly relevant to plasma-derived EV biomarker panels containing proteins involved in lipid transport, complement, and coagulation, such as APOE, C1Q, PLG, and SERPINC1 [25]. Conventional bulk proteomics alone cannot reliably distinguish intravesicular proteins from integral membrane proteins, peripherally associated proteins, protein-corona components, or soluble proteins and protein aggregates co-isolated with EVs [18,24]. Accordingly, unless localization has been demonstrated experimentally, the conservative term “EV-associated protein” is used throughout this review.

The biological effect of an EV-associated protein depends on a sequence of events extending beyond its detection in an isolated EV preparation. These events include EV release by the donor cell, persistence in ascitic fluid or the circulation, interaction with soluble proteins and extracellular matrix components, recognition and adhesion to a recipient cell, vesicle internalization or membrane fusion, and exposure of the active protein to its receptor, substrate, or intracellular signaling machinery [18,23]. The relative importance of these stages depends on protein topology. Surface-accessible EV proteins may act without complete vesicle internalization by directly engaging receptors or extracellular matrix components. This mechanism is consistent with the effects of the exosomal integrin α5β1/asparaginyl endopeptidase complex on mesothelial cells, PKR1-positive OC exosomes on endothelial cells, and PD-L1-positive macrophage-derived exosomes on CD8+ T cells [26,27,28]. In contrast, luminal proteins generally require productive uptake, membrane fusion, or intracellular processing before they can interact with cytoplasmic targets [18]. Several ovarian-cancer studies illustrate distinct stages of this process. OC-derived exosomes transfer CD44 to peritoneal mesothelial cells and increase their susceptibility to tumor-cell invasion [29]. Exosomal ANXA2 induces mesothelial–mesenchymal plasticity through PI3K/AKT/mTOR signaling [30], whereas serum exosome-associated LRP1 activates ERK signaling and increases MMP2 and MMP9 expression in recipient OC cells [31]. In another mechanistically resolved model, laminin-enriched exosomes released by ETS1-overexpressing OC cells were preferentially internalized by integrin αvβ5-positive omental macrophages and promoted AKT/Sp1-dependent M2 polarization and omental metastasis [32]. These studies demonstrate that functional validation may require evidence of target-cell binding or uptake, protein-dependent signaling, and a measurable phenotypic response. Accordingly, proteomic detection alone does not establish extracellular stability, recipient-cell specificity, productive uptake, or functional delivery of an EV-associated protein [18].

Exosomal proteins may contribute to EMT, a process associated with loss of epithelial polarity, increased motility, invasion, and resistance to apoptosis [19]. Proteomic analysis of exosomes secreted by CAOV3 cells during EGF-induced EMT identified PLAU, LAMB1, COL6A1, and TGFB1 as candidate proteins associated with a mesenchymal HGSOC phenotype [33]. These proteins are biologically coherent with dissemination: PLAU participates in proteolytic remodeling and invasion, whereas LAMB1 and COL6A1 are extracellular matrix components potentially involved in adhesion and tissue remodeling [33,34]. LAMB1 has also been identified in chemoresistant OC cells, and its overexpression has been associated with poor prognosis [35,36]. The relevance of exosomal LAMB1 to ovarian cancer dissemination is further supported by a mechanistic study in A2780 and HO-8910 cells. ETS1-overexpressing ovarian cancer cells released exosomes enriched in LAMB1 together with LAMA5 and LAMC1. These laminin-enriched exosomes were preferentially internalized by integrin αvβ5-positive omental macrophages, promoted AKT/Sp1-dependent M2 polarization and CXCL5/CCL2 production, and enhanced omental metastasis in vivo. Because the laminins were investigated as a functional group, the individual contribution of LAMB1 remains to be determined [32]. COL6A1 was identified among proteins enriched in exosomes released by EGF-treated CAOV3 cells and was associated with the transcriptomic profile of mesenchymal HGSOC [33]. Functional OC studies have independently implicated collagen VI in tumor aggressiveness: collagen VI-rich extracellular matrix promotes platinum resistance, while increased collagen VI signaling has been associated with invasiveness, stemness, metastatic capacity, and adaptive chemoresistance in HGSOC models [37,38,39]. However, these studies generally evaluated collagen VI as a heterotrimeric matrix component or focused predominantly on COL6A3 and therefore do not establish a specific functional role for EV-associated COL6A1. In osteosarcoma models, exosomal COL6A1 was shown to be transferred to fibroblasts and to induce a tumor-supportive phenotype, providing cross-cancer evidence for a possible EV-mediated mechanism that remains to be validated in ovarian cancer [40]. Importantly, the EMT-associated EV protein profile reported by Ferreira et al. was derived from a single EGF-treated CAOV3 cell model [33]. Although the identified proteins were compared with transcriptomic characteristics of mesenchymal HGSOC, the reproducibility of this EV signature has not yet been established in additional HGSOC cell lines, patient-derived models, or clinical EV samples. PLAU, LAMB1, COL6A1, and TGFB1 should therefore be regarded as candidate components of an EMT-associated OC EV signature rather than as universally validated markers of mesenchymal HGSOC [33].

Exosomal membrane proteins mediate ligand–receptor interactions and may determine which recipient cells internalize vesicles [23]. This principle is highly relevant for OC because successful peritoneal implantation requires the transformation of the mesothelial surface from a protective barrier into a permissive niche [5]. In this regard, CD44 is one of the earliest mechanistically characterized EV-associated proteins implicated in OC dissemination. It was shown that OC-derived exosomes can transfer CD44 to peritoneal mesothelial cells and increase their susceptibility to invasion by cancer cells [29]. Thus, even before direct tumor-cell attachment, EV-mediated transfer of CD44 may precondition the mesothelium and facilitate secondary implantation [29]. This study provides evidence of protein transfer to recipient cells, whereas the relative contributions of surface receptor engagement and complete vesicle internalization were not separately determined.

Integrins represent another key group of exosomal proteins involved in peritoneal spread. The exosomal integrin α5β1/AEP complex derived from epithelial OC cells was shown to promote peritoneal metastasis by regulating mesothelial cell proliferation and migration [26]. This finding is mechanistically important because it demonstrates that exosomal adhesion proteins are not only markers of tumor-derived EVs, but can directly modify recipient cells within the metastatic niche. Moreover, lineage-informed EV proteomic studies later identified several integrin-associated or adhesion-related surface proteins in HGSOC-derived EVs, including ITGA2, ITGA5, ITGB3, IGSF8, MYOF, ACSL4, and FOLR1 [41]. In plasma-EV assays, combinations such as IGSF8 and ITGA5 showed high discriminatory performance for HGSOC [41]. Although these diagnostic studies were not designed to prove causality in metastasis, the repeated detection of integrins and cell-surface adhesion proteins supports the idea that EV cargo reflects a dissemination-competent phenotype. Single-EV profiling further strengthened this concept. Bulk EV proteomics averages mixed vesicle populations, whereas tumor-derived particles may represent only a minor fraction of circulating EVs. Wu et al. used single-EV proteomic profiling and identified OC-associated EV subpopulations enriched with integrin-related proteins, including ITGB3, ITGB1, and ITGA6, as well as clinically relevant panels including ITGA6, ITGB2, ILK, APOE, and CD44 [42]. ITGB3 is particularly noteworthy, since it was identified both in lineage-informed HGSOC EV studies and in single-EV profiling, suggesting that some adhesion-associated proteins may be reproducible at the level of distinct EV subpopulations [41,42].

Alongside integrins, tetraspanin-enriched exosome subpopulations may also be relevant to the dissemination-associated phenotype of OC. In plasma from OC patients, CD9-positive exosomes contained significantly higher levels of CD151-positive, Tspan8-positive, and CD151/Tspan8 double-positive vesicles than plasma exosomes from healthy females [43]. CD24 expression on CD9-positive exosomes was also significantly increased in OC patient plasma and differed between plasma- and ascites-derived exosomes [43]. These findings are biologically relevant because CD151 and Tspan8 organize tetraspanin-enriched membrane domains capable of coordinating adhesion receptors, proteases, and signaling molecules involved in cellular motility, stromal remodeling, and angiogenesis. Moreover, the level of CD151+/Tspan8+/CD9+ exosomes in ascites correlated with exosomal miR-24-3p levels, suggesting that membrane proteins and regulatory vesicular cargo may be jointly reorganized in dissemination-associated vesicle populations [43]. Although these data do not directly establish a causal role of CD151- or Tspan8-positive exosomes in peritoneal implantation, they identify a clinically detectable OC-associated EV phenotype compatible with increased invasive potential.

A second major axis of EV-associated protein function in OC dissemination involves cell migration, angiogenesis, and extracellular matrix remodeling. Proteomic comparison of exosomes released by ovarian-cancer cell lines with different invasive capacities identified GNA12, EPHA2, and collagen alpha-1(XVIII) chain, encoded by *COL18A1*, among proteins associated with the ability of tumor-derived exosomes to stimulate migration of endothelial and mesenchymal cells [44]. In a separate study, exosomes derived from high-grade OC cell models promoted endothelial-cell proliferation, migration, and tube formation, and proteomic profiling highlighted ATF2, MTA1, and ROCK1/2 as candidate angiogenesis-associated components [45]. CD147/BSG was also detected on exosomes released by the ovarian-cancer cell lines OVCAR3, SKOV3, and A2780. CD147-positive vesicles promoted an angiogenic phenotype and induced matrix-metalloproteinase expression in HUVECs, whereas CD147 depletion reduced the angiogenic activity of the vesicles [46]. These studies indicate that OC-derived EVs may contain proteins associated with cytoskeletal remodeling, cellular migration, matrix degradation, and endothelial activation [44,45,46]. Serum exosomes from patients with epithelial OC contained increased levels of LRP1, a member of the low-density lipoprotein receptor family involved in the regulation of intracellular signaling [31]. Functional experiments demonstrated that exosome-associated LRP1 activated ERK signaling, increased MMP2 and MMP9 expression, and promoted OC-cell migration in vitro and metastatic progression in vivo [31]. This mechanism is relevant to peritoneal dissemination because MMP2 and MMP9 contribute to extracellular matrix degradation and invasion through mesothelial and submesothelial structures. EV-associated LRP1 may therefore represent both a candidate biomarker and a functional mediator of matrix-remodeling-dependent OC dissemination [31].

Patient-derived sEV studies further support an association between vesicle-associated proteases and the extent of peritoneal disease. In advanced OC, CD9-positive sEVs isolated from plasma and ascites contained ADAM10, ADAM17, MMP2, MMP9, and the matrix-metalloproteinase inducer EMMPRIN/CD147 [47]. Although the metalloproteinase composition of plasma-derived CD9-positive sEVs was broadly similar between patients with borderline ovarian tumors and those with OC, ascites-derived sEVs differed in the proportions of ADAM-associated subpopulations [47]. Within the OC cohort, the distribution of ADAM- and MMP/EMMPRIN-positive sEVs depended on ascites volume: the proportions of MMP9+/MMP2+/EMMPRIN+ and MMP9+/MMP2+/EMMPRIN− plasma sEVs were higher in patients with low-volume ascites than in those with moderate- or high-volume ascites [47]. Furthermore, the ADAM10+/ADAM17− subpopulation of plasma-derived CD9-positive sEVs was positively associated with the peritoneal carcinomatosis index [47].

An earlier analysis demonstrated increased ADAM10 expression specifically in the CD24-positive ascites exosome subpopulation of patients with OC, whereas ADAM10 expression in CD9-positive exosome fractions from patients with ovarian tumors was comparatively low [48]. These observations indicate that invasion-associated proteases may be concentrated within particular EV subpopulations and that their detection depends substantially on the surface marker used for vesicle isolation. EV-associated metalloproteinases may therefore represent both mechanistically plausible mediators of extracellular matrix remodeling and candidate indicators of local dissemination burden and ascites-associated microenvironmental changes [47,48]. Annexin A2 is another EV-associated protein with direct relevance to mesothelial reprogramming. Exosomal ANXA2 derived from OC cells was shown to regulate epithelial–mesenchymal plasticity in human peritoneal mesothelial cells [30]. Exosomal ANXA2 promoted migration, invasion, morphological remodeling, fibrosis-associated changes, and mesothelial–mesenchymal transition through activation of the PI3K/AKT/mTOR pathway [30]. These findings support the concept that OC-derived exosomes contribute to dissemination not only through their effects on tumor cells but also by converting normal peritoneal cells into active participants in metastatic-niche formation. However, the study demonstrated an ANXA2-dependent response to the EV preparation without conclusively distinguishing signaling initiated at the cell surface from signaling occurring after vesicle internalization [30].

Exosomal proteins may also support survival of disseminated OC cells in ascites. Detached tumor cells must resist oxidative stress, immune attack, and chemotherapy. Spheroid formation is one of the key adaptive strategies in ascitic fluid. Hypoxia-induced HIF1α-dependent COX2 expression has been shown to promote spheroid formation, inflammatory signaling, and metastatic progression in ovarian cancer cells [49]. Whether COX2 is functionally transported by OC-derived EVs remains to be established. Since hypoxia is a characteristic feature of the tumor microenvironment, hypoxia-driven alterations in exosomal cargo may promote survival of suspended tumor aggregates and increase their ability to implant on the peritoneal surface.

Protease-associated components of OC-derived EV preparations are not limited to membrane-associated metalloproteinases, as increased levels of the 20S proteasome have also been detected in plasma- and ascites-derived exosome fractions from patients with ovarian tumors [20,48]. The 20S proteasome, a tetraspanin-unassociated proteolytic complex, was detected at increased levels in plasma exosomes from OC patients compared with healthy females in an initial study of circulating exosomal proteases [48]. A subsequent investigation demonstrated increased 20S proteasome levels in OC tissue and in plasma exosomes from OC patients, while its abundance in plasma- and ascites-derived exosomes from patients with ovarian tumors was generally comparable [20]. Notably, ascites exosomes from OC patients with low-volume ascites contained significantly higher levels of 20S proteasome than exosomes from patients with moderate- or high-volume ascites [20]. At the same time, chymotrypsin-like and caspase-like proteasomal activities in plasma and ascites exosomes remained below the detection limit, despite the increased abundance of the proteasome protein complex [20]. Therefore, in contrast to surface-associated MMPs and ADAM proteins, exosomal 20S proteasome should currently be interpreted primarily as a cargo signature of tumor-associated vesicles and ascitic disease state rather than as a proven extracellular proteolytic effector of peritoneal invasion. Its enrichment in an enzymatically inactive form may reflect selective intravesicular loading, peripheral association with EVs, or the presence of inhibitory factors within the isolated EV fraction; however, the topology of the EV-associated 20S proteasome has not yet been established [20].

Angiogenesis represents another essential component of OC dissemination, especially after implantation of tumor cells onto the peritoneal surface. Exosomal proteins can activate endothelial cells and promote neovascularization of metastatic implants. A study of tumor-associated exosomes derived from A2780 and HO-8910 cell cultures demonstrated that PKR1-positive exosomes can induce angiogenesis in vitro [27]. When HUVECs were treated with PKR1-positive exosomes, endothelial migration and tube formation increased compared with controls; this effect was associated with phosphorylation of STAT3 [27]. Thus, the PKR1/STAT3 axis may represent one mechanism by which OC-derived exosomes stimulate angiogenic remodeling.

Immune suppression is another major mechanism through which exosomal proteins promote dissemination. The peritoneal cavity in OC contains not only tumor cells, but also tumor-associated macrophages, T-cells, fibroblasts, and other stromal components. PD-L1-positive exosomes released from tumor-associated macrophages were shown to promote peritoneal metastasis of epithelial OC by inducing metabolic dysfunction in CD8+ T cells [28]. In this study, PD-L1+ tumor-associated macrophage (TAM)-derived exosomes altered lipid metabolism through the PPARα–CPT1A axis, increased reactive oxygen species (ROS) accumulation, and promoted CD8+ T-cell exhaustion and apoptosis [28].

Ascitic EVs provide particularly important information about the local metastatic niche. Shender et al. showed that malignant ovarian ascites contains a structured proteome–metabolome communication network, rather than nonspecific inflammatory debris [22]. Quiralte et al. identified and orthogonally validated STX5 and S100A4 in peritoneal-fluid-derived sEVs from patients with ovarian cancer. Their abundance was associated with disease progression, platinum chemosensitivity, and patient outcome [50]. S100A4 has an established functional relationship with ovarian-cancer invasion, EMT, chemoresistance, and metastasis, whereas exosomal S100A4-mediated signaling has been functionally demonstrated mainly in hepatocellular and lung cancer models [51,52,53,54,55,56]. For STX5, the current ovarian-cancer evidence is limited to its enrichment and clinical association in peritoneal-fluid-derived sEVs [50]; its PI3K/mTOR-associated prometastatic function has thus far been investigated in hepatocellular carcinoma [57]. Accordingly, S100A4 can be described as an OC-relevant prometastatic protein detected in sEVs, whereas STX5 should be considered an OC sEV-associated prognostic candidate whose functional role remains to be established [50,51,52,53,54,55,56,57]. Nevertheless, these markers should currently be considered outcome-associated candidates identified in peritoneal-fluid-derived sEVs rather than fully validated functional mediators of OC dissemination.

Therapy resistance is also closely linked to dissemination, recurrence, and persistence of metastatic disease. Platinum-resistant cells are more likely to survive treatment, remain viable in ascites, and seed recurrent lesions. Wagner et al. showed that circulating EV proteins, particularly CFH and TMEM205, may predict platinum resistance in HGSOC [58]. Although this study did not directly prove that CFH- or TMEM205-containing EVs initiate metastasis, the association with platinum-resistant biology is highly relevant to clinically significant dissemination and recurrence-prone disease [58]. CFH may reflect complement-regulatory immune escape, whereas TMEM205 has been linked to therapy resistance mechanisms [58]. In this framework, EV proteins can participate in dissemination research not only as direct metastatic effectors, but also as indicators of aggressive, treatment-selected tumor states.

Taken together, the available data indicate that exosomal proteins participate in OC dissemination through several interconnected mechanisms. Direct mechanistic evidence is strongest for CD44 transfer to mesothelial cells, the exosomal ITGA5/ITGB1/AEP complex, exosomal ANXA2, exosomal LRP1, and PD-L1-positive tumor-associated macrophage exosomes. A broader set of proteomic and clinical studies supports the involvement of adhesion molecules, integrins, EMT-associated proteins, complement/coagulation proteins, immune regulators, and platinum-resistance-associated proteins in OC progression, but many of these findings remain association-level evidence and require functional validation in EV-specific dissemination models.

## 3. Exosomal Proteome as a Source of Markers for Liquid Biopsy of OC

A number of studies have been devoted to the identification of specific proteomic biomarkers in exosomes and other EVs derived from OC cells. One of the first large-scale proteomic maps of OC-derived exosomes was obtained by Liang et al. using OVCAR-3 and IGROV1 cell lines. In this study, more than two thousand proteins were identified, and it was shown that OC-derived EVs are enriched with proteins involved in adhesion, migration, invasion, and metastasis [59]. Later, an in-depth proteomic analysis of EVs from four OC cell lines demonstrated that EVs are not random fragments of parental cells, but selectively enriched vesicular structures whose protein composition differs from that of donor cells in a functionally interpretable manner [60]. In another study, Cheng et al. compared EVs secreted by SKOV-3 cells and non-malignant ovarian surface epithelial cells. A total of 1433 proteins were identified, and several malignant-cell-associated candidates, including COL5A2 and LPL, were proposed; in addition, the authors showed that lipidomic profiling can complement proteomic signatures [61]. Although these studies were not directly aimed at clinical diagnosis, they formed the basis for the concept that proteins transported by OC-derived EVs contain cancer-associated biological information and may be used for the development of liquid biopsy approaches.

Ascites and other local biological fluids provide an additional biologically relevant source of EV-associated proteins in OC. Shender et al. showed that malignant OC ascites contains a structured proteome–metabolome communication network rather than nonspecific inflammatory debris, thereby supporting the rationale for vesicle-focused biomarker studies in this compartment [22]. Barnabas et al. further developed this concept by analyzing vesicle-associated proteins from uterine lavage, which is an anatomically relevant fluid in the context of tubal and serous carcinogenesis. In that study, a nine-protein classifier was proposed, which detected all stage I lesions in the analyzed cohort [62]. Thus, uterine liquid biopsy may be considered an intermediate approach between tissue biopsy and blood-based liquid biopsy: it is more proximal to the presumed site of origin of many HGSOCs than plasma but remains substantially less invasive than surgical sampling [4,62]. Proteomic profiling of peritoneal-fluid-derived sEVs identified STX5 and S100A4 as candidates associated with patient outcome and platinum-response characteristics [50]. Their functional interpretation is discussed in Section 2. Of note, it was demonstrated that the proteome of ascitic EVs is not exclusively tumor-cell-derived but contains substantial stromal and immune-cell contributions, particularly from macrophages and fibroblasts [63]. This observation is diagnostically important because it complicates a simplified “tumor-marker only” interpretation of EV cargo, but at the same time broadens the biomarker concept: clinically informative EV signatures in OC may include both tumor-derived and microenvironment-derived proteins, since both components participate in peritoneal dissemination.

The clinical translation of EV proteomics in OC became more active when discovery studies were followed by blood-based case–control designs. Zhang et al. analyzed plasma exosomes using iTRAQ-LC-MS/MS and proposed a five-protein panel consisting of APOE, EpCAM, PLG, SERPINC1, and C1Q, which demonstrated an AUC of 0.913 for epithelial OC detection [25]. Their diagnostic association may therefore reflect proteins loaded into EVs by donor cells, stable peripheral or corona association with circulating EVs, or reproducible co-isolation with particular EV populations [18,24]. This work remains important because the proposed panel combined epithelial/tumor-associated proteins with host-response proteins involved in coagulation, complement activation, and lipid transport [25]. A more lineage-oriented strategy was later used by Trinidad et al., who selected candidate EV surface proteins using fallopian-tube and HGSOC tissue explants, cell lines, and plasma, thereby linking biomarker discovery to the accepted tubal origin model of HGSOC [41]. The proposed candidate set included ACSL4, IGSF8, ITGA2, ITGA5, ITGB3, MYOF, and FOLR1; moreover, an ExoProfile-based combination of IGSF8 and ITGA5 showed AUC values approaching 0.99 in the analyzed dataset [41]. Targeted proteomic approaches subsequently began to bridge discovery proteomics and assay development. Cooper et al. combined DDA, DIA, targeted PRM, and ELISA validation using EVs from cell lines, ascites, and plasma, and repeatedly prioritized MUC1-containing EV signatures for early-stage HGSOC detection [64]. Importantly, this study did not propose a single universal diagnostic panel, but showed that MUC1 may serve as a central component of several panel configurations, including combinations with APOC4, CFHR4, and GPX3 [64]. This suggests that the most reproducible diagnostic signal may be associated with MUC1-containing EV signatures rather than with a single fixed multivariate model. Rayamajhi et al. further developed this direction by analyzing circulating small EV proteins and proposing a compact four-protein panel, including MUC1, MYL6, TTYH3, and GSTP1, for early-stage HGSOC detection, with a reported AUC of 0.975 against healthy controls [65]. Lightfoot et al. expanded the spectrum of candidate serum EV proteins and prioritized AGRIN, CFH, PZP, CCNE1, FAS, SPP24, STAT3, PD-L1, and IL6 as components of a high-performing retrospective panel for early-stage HGSOC [66]. Taken together, these studies indicate that recurrent blood-based EV protein signals in OC can be divided into several main biological groups: epithelial/tumor-lineage proteins, adhesion and integrin-associated surface molecules, and systemic immune–complement–coagulation proteins.

Single-EV analysis represents an emerging direction in the development of this field. Bulk EV proteomics averages signals across mixed vesicle populations, which is a serious limitation for early-stage OC, where tumor-derived EVs may represent only a minor fraction of all circulating particles. In this regard, modern EV methodology increasingly focuses on single-particle phenotyping, which makes it possible to distinguish tumor-enriched EV subpopulations from the surrounding plasma background. In OC, single-EV proteomic profiling based on proximity barcoding and machine learning was applied and a diagnostic panel including ITGA6, ITGB2, and ILK was proposed [42]. In addition, the authors identified a tumor-associated EV subpopulation enriched with ITGB3, ITGB1, and ITGA6 [42]. This finding is clinically relevant because ITGB3, previously highlighted in lineage-informed HGSOC surface-marker studies, may be more reproducible at the level of defined EV subpopulations than in bulk EV isolates [41,42]. Candidates were included in Table 1 if they had been evaluated in human ovarian-cancer-derived EV samples and proposed as diagnostic, prognostic, or treatment-response biomarkers; proteins supported exclusively by mechanistic cell-line experiments were not included unless they were also assessed in clinical EV samples.

Protein topology was not established in all cited studies. Accordingly, the listed candidates may include surface-accessible proteins, intravesicular components, peripherally associated proteins, or protein-corona components.

Protein and miRNA profiling of OC-associated EVs should be regarded as complementary rather than competing liquid-biopsy strategies. EV-associated miRNAs can reflect regulatory programs operating in tumor and stromal cells and may exert functional effects after transfer to recipient cells. For example, stromal-cell-derived exosomal miR-21 was shown to suppress *APAF1* expression and increase paclitaxel resistance in ovarian cancer cells [67]. In addition, the abundance of miR-24-3p in ascites-derived exosomes correlated with the proportion of CD151+/Tspan8+/CD9+ vesicles, indicating that regulatory RNAs and surface-protein phenotypes may be coordinately represented within clinically relevant EV populations [43]. From an analytical perspective, miRNAs offer high sensitivity because nucleic-acid targets can be amplified, but their measurement is strongly influenced by low copy number, RNA extraction efficiency, hemolysis, normalization strategy, and contamination by non-vesicular RNA–protein or lipoprotein complexes [18]. EV protein analysis does not benefit from target amplification and is affected by the wide dynamic range of plasma proteins and by possible co-isolation of soluble or corona-associated proteins [18,24]. However, proteins provide several advantages that are particularly relevant to EV-based diagnostics. Surface-accessible proteins can be used for immunocapture, phenotyping, and enrichment of tumor-associated EV subpopulations, while proteomic and single-EV approaches can simultaneously evaluate epithelial markers, integrins, immune regulators, and host-response proteins [25,41,42,64,65,66]. Moreover, membrane-associated proteins may act directly on recipient cells without requiring release of luminal cargo, making protein profiling informative not only for biomarker discovery but also for the investigation of EV-mediated mechanisms of dissemination [26,28,29,30]. Therefore, combined analysis of EV surface proteins and regulatory RNAs may provide greater biological and diagnostic resolution than either analyte class alone. Protein markers can help identify the cellular origin and surface phenotype of individual EV populations, whereas miRNAs may provide information about regulatory activity and functional reprogramming. Future OC liquid-biopsy studies should therefore evaluate integrated protein–miRNA signatures within the same EV fractions or, where technically feasible, within defined EV subpopulations [18,43].

Collectively, EV-proteomic studies have identified candidate OC biomarkers in plasma, serum, ascites, peritoneal fluid, and uterine lavage. The most recurrent signals include epithelial and lineage-associated proteins, integrins and other surface molecules, MUC1-centered signatures, and host-response proteins involved in complement, coagulation, immunity, and treatment resistance [25,41,42,62,64,65,66]. These findings support the development of multianalyte EV panels, but prospective validation, standardized EV isolation, and independent clinical cohorts remain necessary.

## 4. Exosomal Proteome as a Source of Information on the Functional and Metabolic Status of OC

Because exosomal cargo reflects the cellular source and selective cargo sorting, the exosomal proteome may be used not only for diagnostic research but also to characterize biological processes represented in tumor-derived vesicles [5,19,68]. Therefore, we performed a bioinformatic analysis to evaluate the representation of functional and metabolic pathways among proteins identified in OC-associated exosomes, described in the studies above.

**Figure 1:**
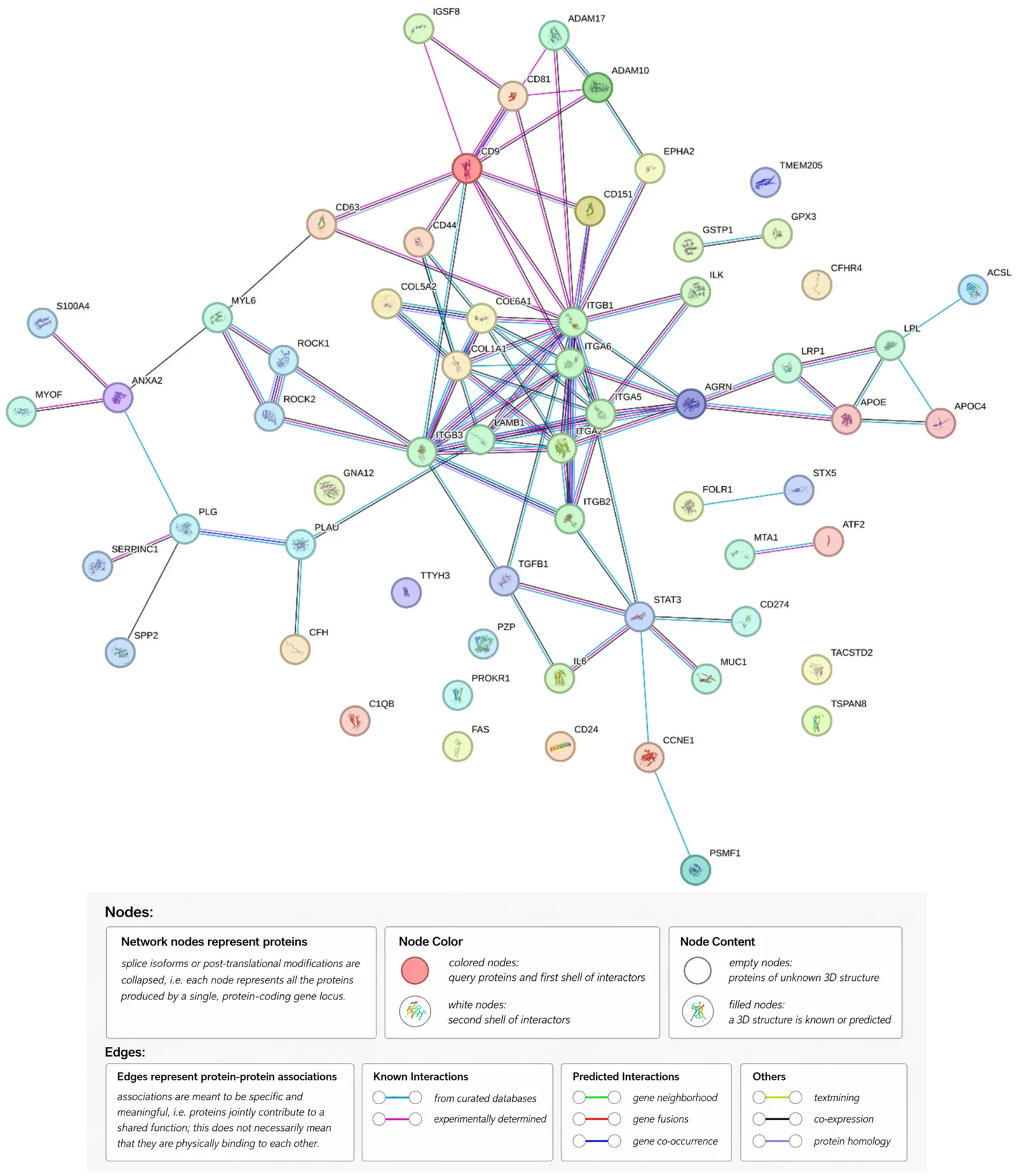
STRING protein–protein association network of the selected ovarian-cancer-associated EV proteins. The network was generated for Homo sapiens using STRING version 12.0 with a minimum required interaction score of 0.400, corresponding to medium confidence. Text-mining evidence was excluded, no additional first- or second-shell interactors were added, and the network was restricted to the submitted protein set. Nodes represent proteins, whereas edges represent STRING-supported functional or physical protein associations; edge colors indicate the supporting evidence channels. Disconnected proteins were retained. The network was generated using STRING version 12.0.

**Figure 2:**
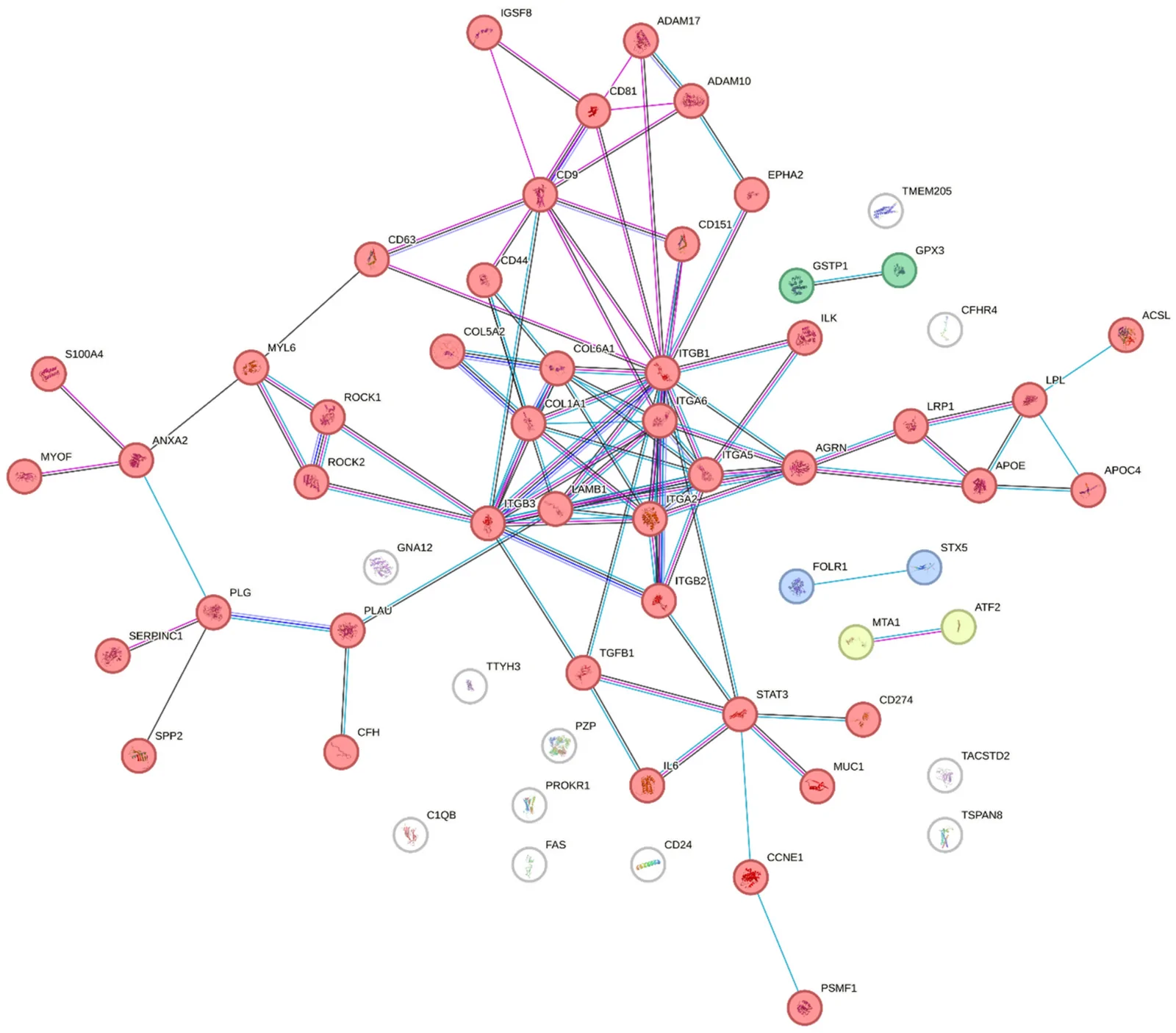
K-means clustering of the STRING protein–protein association network of ovarian-cancer-associated EV proteins. The submitted protein set was partitioned into four clusters using the built-in STRING k-means procedure with *k* = 4. Nodes represent proteins, and edges represent STRING-supported protein associations. Cluster colors are as follows: red, integrin and cell-surface interaction-associated cluster; green, reactive-oxygen-species detoxification-associated cluster; blue, endoplasmic-reticulum cargo concentration and secretory-trafficking-associated cluster; and yellow, the ATF2/MTA1-associated cluster. The network was generated using STRING version 12.0.

**Figure 3:**
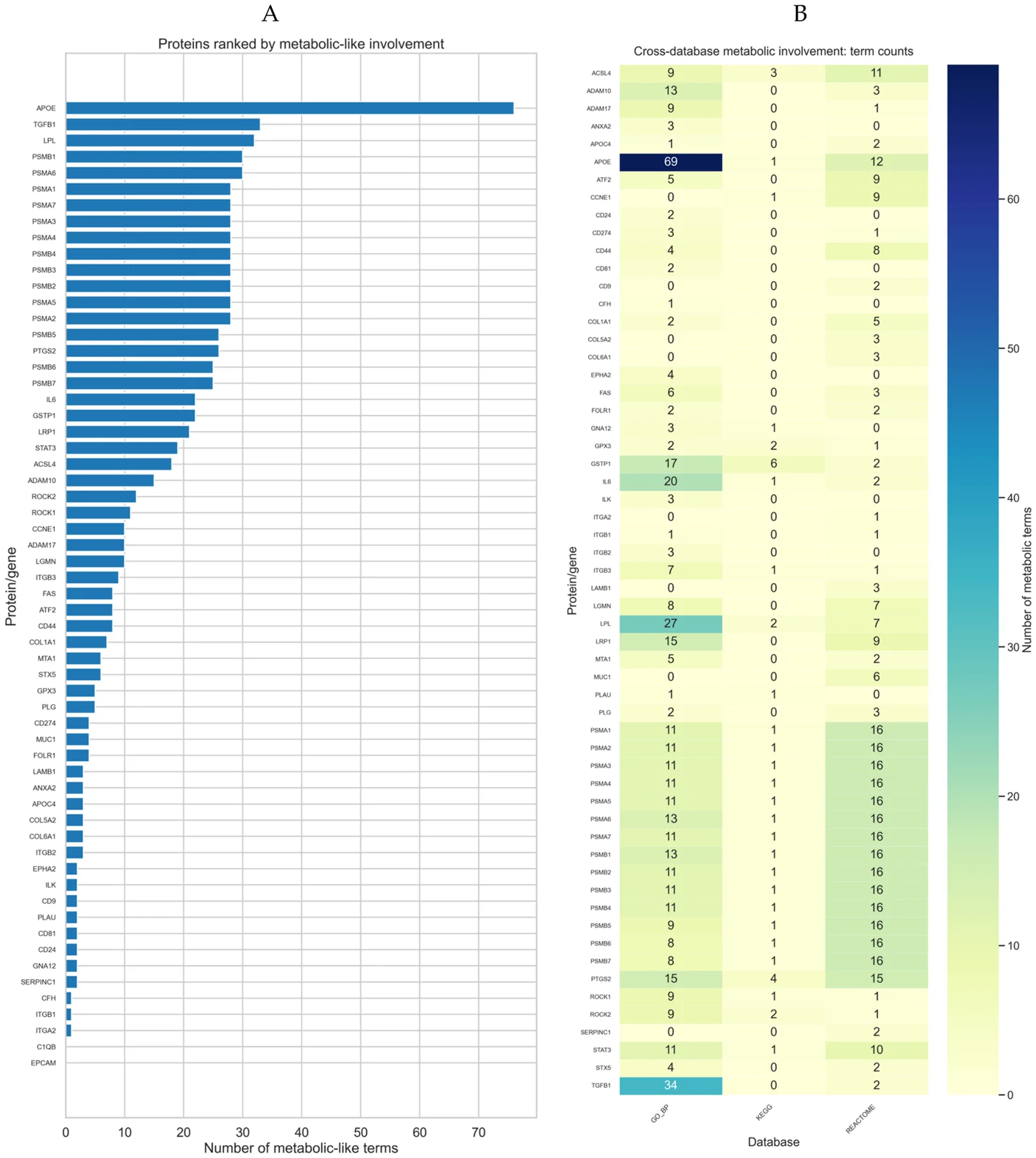
Annotation coverage of the selected ovarian-cancer-associated EV proteins across functional and pathway resources. (**A**) Total number of metabolic and metabolism-associated functional terms assigned to each protein across the analyzed Gene Ontology Biological Process, KEGG, and Reactome libraries. (**B**) Heatmap showing the number of Gene Ontology Biological Process, KEGG, and Reactome terms assigned to each protein. Numbers within the cells indicate annotation counts; zero indicates that the protein was not assigned to any term selected by the predefined metabolism-associated keyword filter in the corresponding resource. The color scale ranges from lower to higher annotation counts. The figure was generated by the authors using Python 3.12.12, pandas 3.0.3, Matplotlib 3.10.9, and seaborn 0.13.2. Annotation-coverage counts are provided in the Table S2.

**Figure 4:**
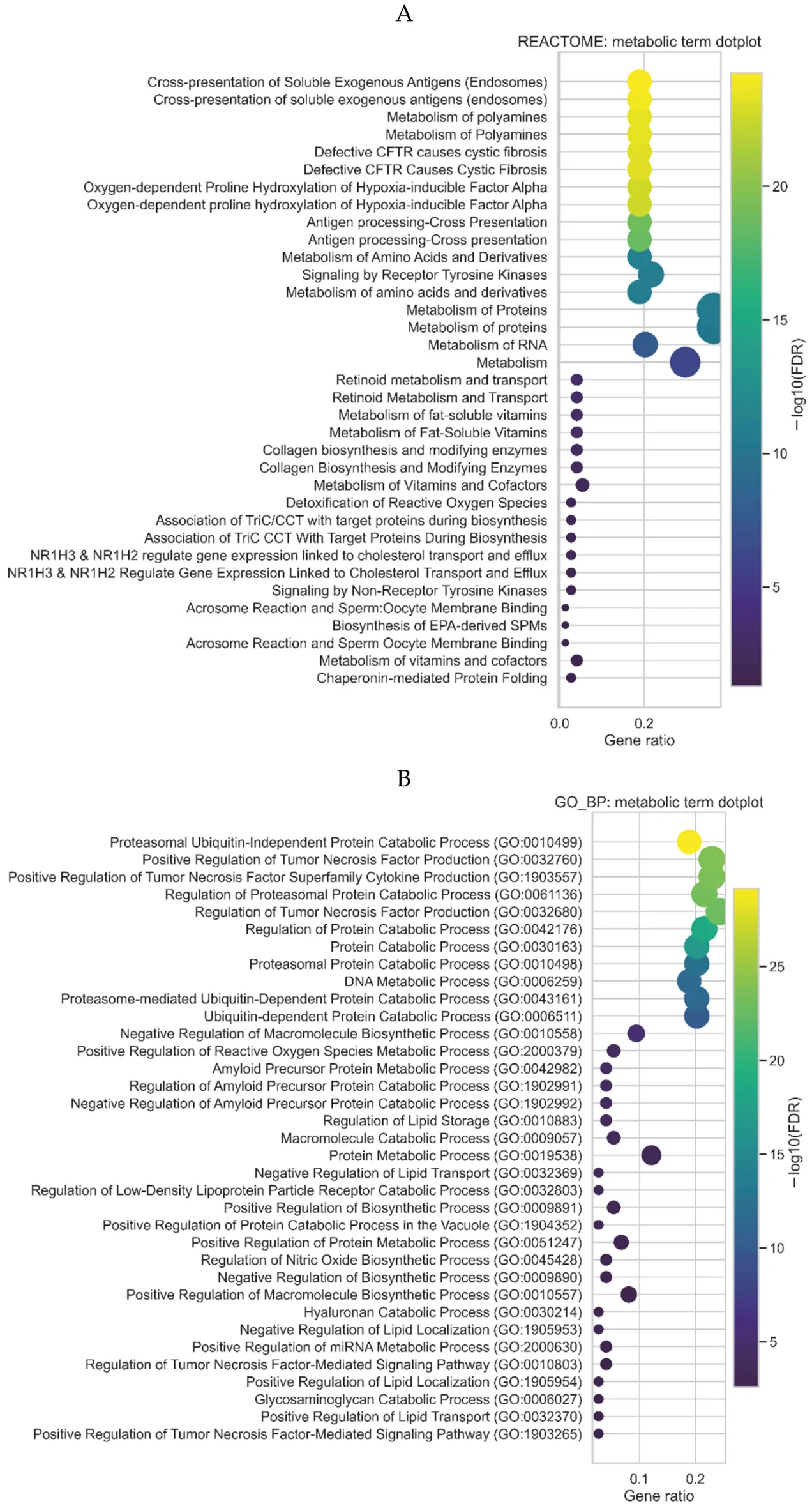
Functional enrichment analysis of proteins reported in ovarian-cancer-associated EV preparations. (**A**) Reactome pathway enrichment. (**B**) Gene Ontology Biological Process enrichment. Over-representation analysis was performed using a one-sided hypergeometric test, and nominal *p* values were adjusted using the Benjamini–Hochberg procedure. The plots show up to 35 of the highest-ranked metabolic and metabolism-associated terms selected using the predefined keyword-based filter. The horizontal axis represents the gene ratio, calculated as the number of input genes assigned to a term divided by the total number of input genes mapped to the corresponding annotation background. Point size indicates the number of overlapping input genes, and point color represents −log10 of the Benjamini–Hochberg-adjusted *p* value. The figure was generated by the authors using Python 3.12.12, pandas 3.0.3, SciPy 1.17.1, Matplotlib 3.10.9, and seaborn 0.13.2. The complete GO Biological Process and Reactome over-representation results, including term identifiers, term sizes, overlap sizes, gene ratios, background ratios, fold-enrichment values, nominal *p*-values, Benjamini–Hochberg-adjusted *p*-values, and contributing genes, are provided in Supplementary Table S3.

**Figure 5:**
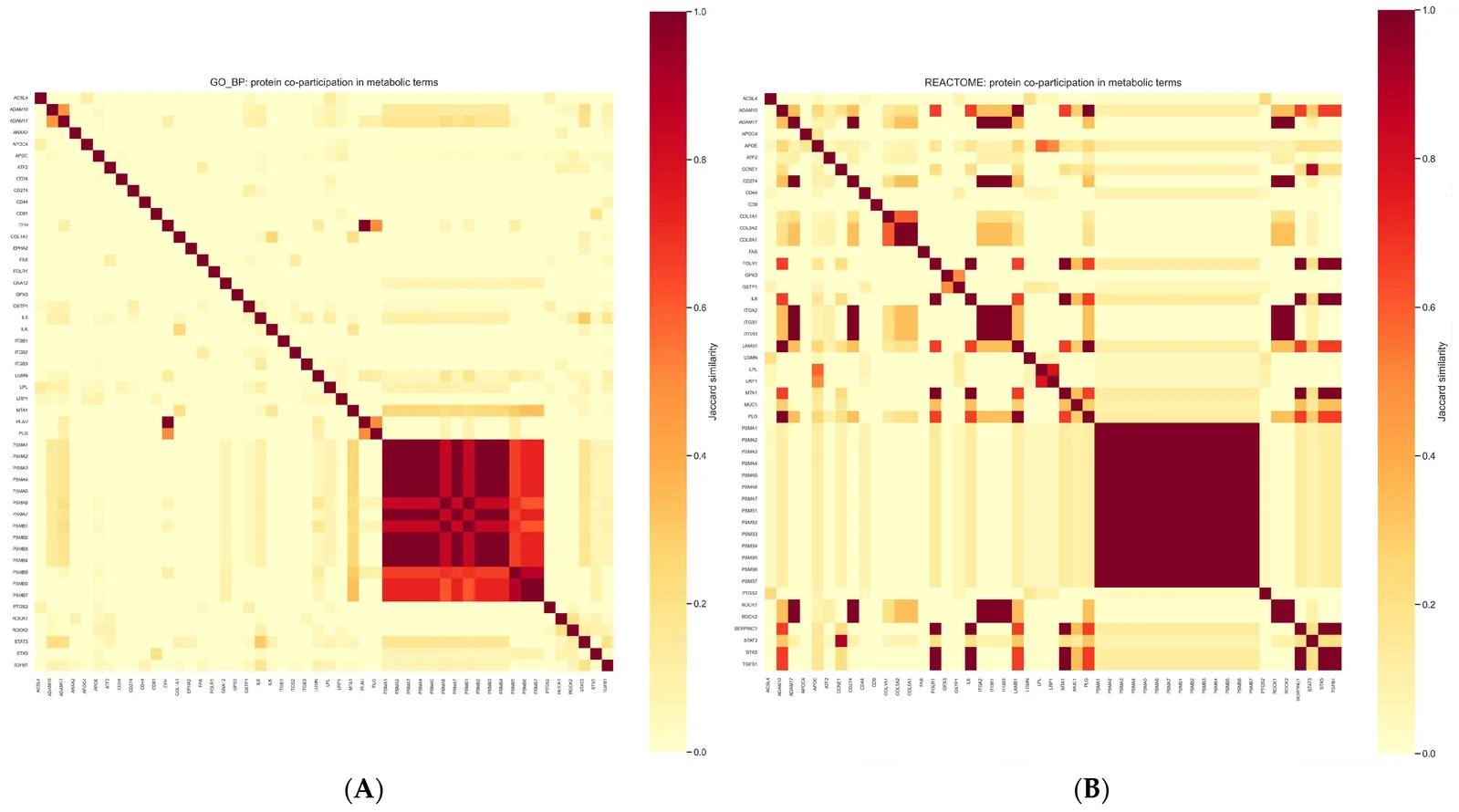
Pairwise similarity of functional annotation profiles among ovarian-cancer-associated EV proteins. (**A**) Jaccard similarity matrix based on Gene Ontology Biological Process terms. (**B**) Jaccard similarity matrix based on Reactome terms. Binary term-by-protein matrices were constructed from the selected metabolic and metabolism-associated annotations, and pairwise similarity was calculated using the Jaccard coefficient. Values range from 0, indicating no shared annotations, to 1, indicating identical annotation sets within the terms included in the analysis. Proteins without assignments to the selected terms were excluded from the corresponding matrix. Jaccard similarity reflects shared functional annotation and does not indicate direct physical interaction, co-localization within the same EV, correlated abundance, or simultaneous pathway activation. The figure was generated by the authors using Python 3.12.12, pandas 3.0.3, SciPy 1.17.1, Matplotlib 3.10.9, and seaborn 0.13.2. Pairwise Jaccard similarity matrix is provided in the Table S4.

Bioinformatic analysis was performed on a manually curated set of 58 EV-associated protein entities reported in the ovarian cancer studies discussed in this review. Proteins were included if they had been explicitly detected in EV preparations derived from ovarian-cancer cell lines or clinical biological fluids, or if they had been investigated as EV-associated functional mediators, diagnostic or prognostic candidates, or markers of treatment response. The complete list of the 58 protein entities, together with their standardized gene symbols, representative source studies, biological materials, reasons for inclusion, functional categories, and evidence levels, is provided in Supplementary Table S1. Protein names and aliases were standardized to human gene symbols before analysis; the C1Q complex was represented by its *C1QA*, *C1QB*, and *C1QC* subunits. Common protein names and aliases were converted to approved human gene symbols: AEP was mapped to *LGMN*, CD147 to *BSG*, COX2 to *PTGS2*, EpCAM to *EPCAM*, PD-L1 to *CD274*, and PKR1 to *PROKR1*. C1Q was retained as one protein entity in the curated dataset but was represented by *C1QA*, *C1QB*, and *C1QC* in gene-based analyses; consequently, the 58 protein entities corresponded to 60 non-redundant human gene symbols. Protein–protein association analysis was performed for Homo sapiens using STRING version 12.0 according to the standard STRING workflow, with a medium-confidence minimum required interaction score of 0.400, with text-mining evidence excluded, and the resulting network was partitioned using k-means clustering with *k* = 4 [69]. Functional annotation and enrichment analyses were performed in Python 3.12.12 using NumPy 2.4.6, pandas 3.0.3, SciPy 1.17.1, Matplotlib 3.10.9, and seaborn 0.13.2. The most recent gene-set libraries available at the time of analysis in May 2026 were used: GO Biological Process 2026 and KEGG 2026 obtained in Enrichr-compatible format, together with the current Reactome pathway GMT release and the Enrichr-compatible Reactome Pathways 2024 library [70,71,72,73]. Over-representation analysis was conducted using a one-sided hypergeometric test; terms containing fewer than 5 or more than 5000 genes were excluded, and the statistical background was defined as the union of human genes represented in the analyzed annotation libraries. Nominal *p* values were adjusted for multiple testing using the Benjamini–Hochberg procedure. Annotation coverage was evaluated across GO Biological Process, KEGG, and Reactome, whereas detailed enrichment analysis was restricted to GO Biological Process and Reactome, which provided the broadest coverage of the protein set. Metabolic and metabolism-associated terms were identified using a predefined keyword-based filter and ranked by the Benjamini–Hochberg-adjusted *p* value; the 35 highest-ranked terms were used for visualization. To evaluate functional co-annotation among individual proteins, binary term-by-protein matrices were constructed separately for GO Biological Process and Reactome, and pairwise similarity between protein annotation profiles was calculated using the Jaccard coefficient. Only the PPI network, STRING k-means clustering, annotation-coverage analysis, GO Biological Process and Reactome enrichment, and protein co-annotation analyses presented in Figure 1, Figure 2, Figure 3, Figure 4 and Figure 5 were included in the manuscript.

We first performed a protein–protein association analysis using STRING (https://string-db.org) (Figure 1).

The PPI analysis revealed a dense association network among most of the analyzed proteins. PPI enrichment test. The STRING network was further partitioned using the built-in k-means procedure with *k* = 4, and the resulting clusters were interpreted according to their principal proteins and functional annotations (Figure 2).

The integrin and cell-surface interaction-associated cluster was consistent with the prominent representation of adhesion proteins in OC EV studies. Integrin-containing EVs may contribute to mesothelial-cell interaction, extracellular matrix recognition, and peritoneal implantation, as supported by functional studies of the exosomal ITGA5/ITGB1/AEP complex and by clinical and single-EV detection of multiple integrin-associated proteins [26,41,42]. Integrin signaling in OC is connected with FAK/Src, PI3K/AKT, and MAPK/ERK pathways involved in invasion, survival, and treatment resistance [74,75,76]. However, the limited efficacy of anti-integrin monotherapy indicates that these proteins are components of a broader dissemination network rather than isolated therapeutic targets [77].

The ATF2/MTA1-associated cluster may reflect transcriptional and stress-response programs represented in OC-derived EVs. MTA1 promotes EMT-associated and angiogenic phenotypes in ovarian cancer, whereas ATF2 is associated with aggressive disease and poor prognosis [78,79]. Both proteins were identified in exosomes from high-grade OC cell models associated with endothelial proliferation, migration, and tube formation [45]. However, direct functional transfer of EV-associated ATF2 or MTA1 to recipient cells has not been demonstrated; this cluster should therefore be regarded as hypothesis-generating.

The reactive-oxygen-species detoxification-associated cluster indicates representation of antioxidant and redox-adaptation proteins in the selected EV set. NRF2-dependent antioxidant responses and extracellular GPX3 activity have been linked to OC survival, stemness, metastatic progression, and platinum resistance [80,81,82]. Because the analysis was based on protein presence rather than quantitative EV cargo, this cluster indicates potential redox-related functional representation rather than demonstrated EV-mediated redox buffering.

The endoplasmic-reticulum cargo concentration and secretory-trafficking-associated cluster may reflect the increased secretory and proteostatic demands of OC cells producing extracellular matrix proteins, membrane receptors, and EV-associated components. ER-stress proteins, including GRP78, PDI, PERK, and ATF6, are elevated in ovarian carcinoma, and GRP78/PDI expression has been associated with poor outcome in HGSOC [83]. Nevertheless, this cluster indicates an association with secretory-pathway annotations and does not establish a direct mechanistic connection between ER cargo concentration and EV biogenesis.

For further analysis, we searched for annotations of the identified proteins in three functional and pathway resources: Gene Ontology (GO), Kyoto Encyclopedia of Genes and Genomes (KEGG), Reactome using Python [71,72,73]. Nineteen of the 58 protein entities were not assigned to any of the metabolic or metabolism-associated terms selected from the three analyzed resources (Figure 3A). GO Biological Process and Reactome provided the broadest annotation coverage and were therefore selected for detailed enrichment and co-annotation analyses (Figure 3B). Figure 3 presents descriptive annotation-coverage counts across Gene Ontology Biological Process, KEGG, and Reactome. Therefore, no inferential statistical test or term-specific *p*-value was applied to this analysis. The complete per-protein annotation counts underlying Figure 3 are provided in Supplementary Table S2.

To further characterize the biological significance of proteins identified in OC-associated exosomes, functional enrichment analysis was performed using GO Biological Process and Reactome annotations. Since the analyzed protein set was compiled from studies of EV-associated proteins, the identified terms should be interpreted as metabolic and signaling processes represented in EV preparations rather than as evidence of intravesicular protein localization or direct pathway activation in tumor or recipient cells. Nevertheless, the enrichment profile revealed several functionally interconnected groups of processes that are highly relevant to OC progression and dissemination, including lipid metabolism, extracellular matrix remodeling, receptor tyrosine kinase signaling, oxidative stress adaptation, inflammatory mediator production, and regulation of RNA-associated processes (Figure 4).

GO Biological Process and Reactome analyses showed representation of lipid storage, transport, fatty-acid metabolism, cholesterol efflux, and fat-soluble-vitamin-associated terms (Figure 4). This pattern is relevant to the preferential colonization of the lipid-rich omentum by OC cells. Omental adipocytes supply fatty acids to metastatic cells, while adipocyte-induced CD36 expression supports tumor growth and peritoneal dissemination [84,85]. The lipid-associated enrichment profile may therefore reflect metabolic adaptation of EV-producing tumor or microenvironmental cells to the omental niche; however, it does not demonstrate direct metabolic activity of the EVs themselves. In addition to lipid-associated processes, both enrichment analyses demonstrated representation of extracellular matrix-related pathways. The GO BP analysis revealed terms associated with hyaluronan metabolism and catabolism, glycosaminoglycan catabolism, and regulation of collagen biosynthesis, whereas the Reactome analysis identified collagen biosynthesis and modifying enzymes, crosslinking of collagen fibrils, and hyaluronan metabolism. These processes are consistent with the extracellular matrix remodeling required for transcoelomic OC dissemination and peritoneal implantation [3]. It was previously shown that collagen-rich omentum acts as a premetastatic niche for OC cells, and that interaction of integrin α2 with collagen promotes adhesion, migration, mesothelial clearance, and formation of peritoneal metastases [86]. Furthermore, hypoxia within the tumor–mesothelial niche increases collagen I deposition and promotes LOX-dependent collagen remodeling, whereas inhibition of collagen crosslinking decreases metastatic tumor burden [87]. The potential contribution of hyaluronan-related processes is also biologically supported, since OC cells can bind hyaluronic acid through CD44, thereby facilitating their attachment to peritoneal surfaces [88]. Consequently, the enrichment of collagen- and hyaluronan-associated terms among OC exosomal proteins may be associated with mesothelial adhesion, extracellular matrix remodeling, and implantation of disseminated tumor cells. Receptor tyrosine kinase signaling was one of the most prominent Reactome categories. This pathway is relevant to OC because c-MET and AXL promote invasion, survival, ascites formation, and metastatic implantation in experimental models [89,90]. Its representation in the EV-associated protein set suggests that EV profiles may reflect invasive signaling states, but interpretation requires consideration of the individual proteins contributing to the enrichment term.

Processes associated with oxidative stress and redox regulation were also represented in both analyses. GO BP terms included positive regulation of reactive oxygen species metabolic process and regulation of nitric oxide biosynthetic process, whereas Reactome analysis identified Detoxification of Reactive Oxygen Species. The simultaneous representation of ROS generation and ROS detoxification is biologically plausible in metastatic OC [5]. Moderate ROS accumulation can promote invasive signaling and matrix remodeling, whereas antioxidant protection is required for survival of detached cells and metastatic implants in ascitic and omental environments. It was demonstrated that OC cells colonizing the omentum experience increased oxidative stress and upregulate G6PD, a key enzyme supporting NADPH production and cellular redox balance; inhibition of G6PD decreased the number of omental metastases in vivo [91]. Furthermore, extracellular glutathione peroxidase GPx3 was shown to support HGSOC progression by protecting tumor cells from extracellular oxidative stress in the metastatic microenvironment [92]. ROS accumulation can also activate the HIF-1α/LOX/E-cadherin axis, which is associated with invasive behavior and metastatic progression of OC cells [93]. Thus, the enrichment of ROS-related terms may reflect redox plasticity of the disseminating tumor compartment, allowing OC cells to exploit ROS-dependent signaling while maintaining protection from oxidative damage. Another important group of represented processes was associated with inflammatory cytokines and lipid mediators. The GO BP dotplot included regulation of tumor necrosis factor production, positive regulation of tumor necrosis factor production, positive regulation of tumor necrosis factor superfamily cytokine production, and prostaglandin metabolic process. Inflammatory mediator-related terms included regulation of TNF production, prostaglandin metabolism, and Reactome pathways associated with prostaglandin and thromboxane synthesis. TNF-α generates an autocrine cytokine and angiogenic network in epithelial OC cells [94], while sustained adrenergic signaling promotes metastatic progression through the ADRB2–NF-κB–PTGS2/PTGES–PGE2 axis [95]. Chemotherapy-induced cytokine and lipid-mediator production can also support tumor growth and ascites formation [96]. These annotations may therefore reflect inflammatory support of the peritoneal niche, although specialized pro-resolving mediator terms may represent compensatory rather than exclusively protumorigenic responses. The GO BP analysis also identified positive regulation of miRNA metabolic process. This term is of particular interest in the context of exosomal cargo, since EVs participate in the transport of regulatory RNA molecules between tumor and stromal cells. It was previously shown that tumor-associated adipocytes and fibroblasts from the omental microenvironment transfer miR-21 to OC cells through exosomes. This transfer suppresses *APAF1* expression and increases resistance of tumor cells to paclitaxel [67]. However, since the current enrichment analysis was based on proteins rather than on small RNAs, the presence of this GO term should not be interpreted as direct evidence for the transport of specific miRNAs. More cautiously, it suggests that the exosomal protein set may contain proteins involved in miRNA biogenesis, processing, sorting, or stability, thereby linking the proteomic profile with regulatory RNA-mediated interactions in the tumor microenvironment.

Reactome analysis also revealed terms associated with retinoid metabolism and transport, metabolism of fat-soluble vitamins, and vitamin D metabolism. These processes may be connected with regulation of differentiation, proliferation, epithelial plasticity, and tumor–microenvironment interactions. For example, all-trans retinoic acid has been shown to exert antitumor effects in serous OC models and to influence invasive properties of tumor cells, including an Annexin A2/S100A10-associated phenotype [97]. Nevertheless, compared with lipid metabolism, extracellular matrix remodeling, ROS adaptation, and inflammatory signaling, the relationship between retinoid or vitamin D metabolism and peritoneal dissemination remains less directly established. Accordingly, these processes should currently be considered hypothesis-generating modules requiring further validation at the level of the individual exosomal proteins contributing to enrichment.

Some of the enriched terms require particularly cautious interpretation. The GO BP term related to regulation of amyloid precursor protein catabolic process has no established central role in OC peritoneal metastasis and may arise from proteins involved in membrane trafficking or vesicle sorting. Similarly, Reactome categories associated with protein folding, chaperonin-mediated protein folding, and association of TriC/CCT with target proteins may reflect increased proteostatic demand in malignant cells rather than dissemination-specific mechanisms. Terms related to acrosome reaction and sperm:oocyte membrane binding are likely to result from shared proteins participating in membrane fusion, adhesion, or exocytosis and should not be interpreted as a tumor-specific biological program. Moreover, several Reactome categories are represented by closely related or duplicated labels differing only by capitalization or annotation hierarchy, including Metabolism of Proteins/metabolism of proteins, Retinoid metabolism and transport/Retinoid Metabolism and Transport, and Collagen biosynthesis and modifying enzymes/Collagen Biosynthesis and Modifying Enzymes. Therefore, these terms should be interpreted as functional modules rather than independent biological events.

Overall, enrichment analysis indicated representation of lipid adaptation, extracellular matrix remodeling, receptor-associated signaling, redox regulation, and inflammatory mediator production within the selected OC EV protein set. These categories correspond to established features of peritoneal dissemination but should be interpreted as functional annotations of a curated protein set rather than evidence of direct pathway activity within EVs.

To further refine the interpretation of metabolic processes represented in the proteome of OC-associated exosomes, we evaluated the co-participation of individual proteins in GO Biological Process and Reactome functional terms and metabolic processes using Jaccard similarity analysis. In contrast to enrichment dotplots, which indicate the functional categories represented in the total protein set, the heatmaps demonstrate the extent to which individual proteins share overlapping functional annotations. Therefore, the detected similarities should not be interpreted as evidence of direct protein–protein interaction, co-localization within the same exosomal particle, or simultaneous activation of these proteins in recipient cells. Nevertheless, the analysis makes it possible to distinguish several functionally coherent protein modules that are biologically relevant to ovarian cancer progression and peritoneal dissemination (Figure 5).

In addition to the matrix-remodeling processes described above, the Reactome heatmap suggests a relationship between adhesion-associated proteins and mesothelial reprogramming. In particular, the presence of integrin-related proteins is consistent with previous observations that mesothelial cells exposed to OC cells secrete fibronectin, which promotes early tumor-cell adhesion and invasion through the integrin α5β1 axis [98]. Thus, the detected integrin-associated co-participation pattern may reflect not only interaction with extracellular matrix components, but also communication between tumor cells and the mesothelial surface during early peritoneal implantation. The GO BP heatmap also showed a pronounced similarity involving CFH and PLG. This pattern may indicate the convergence of complement regulation and proteolytic remodeling in the peritoneal microenvironment. The urokinase/plasminogen activation system has been associated with OC dissemination: increased levels of uPA, uPAR, and their inhibitors were detected in omental and lymph-node metastases compared with primary ovarian tumors [99], and the uPA/PAI-1 system was associated with disease progression and prognosis [100]. In parallel, ovarian tumor cells were shown to secrete complement inhibitors factor H and factor H-like protein, which were also detected in ascitic fluid and may protect disseminated tumor cells from complement-mediated damage [101]. Therefore, the CFH–PLG-associated pattern may reflect two complementary features of metastatic progression: extracellular proteolysis and immune evasion. A further functionally relevant group included IL6, STAT3, and TGFB1. Malignant ascites containing elevated IL-6 has been shown to enhance migration and invasion of OC cells through JAK2/STAT3 signaling [102]. Moreover, increased STAT3 expression in ascites-associated OC cells promotes invasion and metastatic progression [103]. The presence of TGFB1 in the same functional context is also consistent with mesothelial-to-mesenchymal transition, which contributes to formation of a permissive peritoneal niche for OC implantation [104]. Thus, this module may represent an inflammation- and stromal-remodeling-associated component of the exosomal protein profile. Finally, GSTP1 and EPHA2 should be interpreted as proteins associated with aggressive tumor properties rather than as validated EV-mediated dissemination factors. GSTP1 directly influences platinum chemosensitivity in ovarian tumor cells and may therefore reflect detoxification and therapy-resistant phenotypes [105]. EPHA2 overexpression promotes ovarian cancer growth and is associated with an aggressive tumor phenotype [106]. Their presence in the co-participation analysis may indicate clinically relevant features of OC-derived EV cargo, but additional experiments are required to determine whether their exosomal transfer contributes directly to peritoneal spread.

The co-annotation heatmaps further identified overlapping functional profiles related to adhesion, proteolysis, complement regulation, inflammatory signaling, and treatment-associated tumor aggressiveness. Jaccard similarity reflects shared database annotations rather than physical interaction, co-occurrence within individual EVs, or coordinated activity in recipient cells. Because the input proteins were selected from OC EV studies focused on biomarkers, dissemination, and treatment response, the analysis was inherently enriched for cancer-relevant functions and should be regarded as an integrative organization of published evidence rather than an unbiased discovery analysis.

## 5. Conclusions

Exosomal proteins represent a promising source of OC biomarkers because EV protein profiles may reflect both tumor-cell-intrinsic features and interactions of malignant cells with stromal, immune, and mesothelial components of the peritoneal microenvironment. The available studies indicate that recurrent EV-associated proteins, including MUC1, EpCAM, FOLR1, integrins and complement- or immune-related proteins, may form the basis for the development of multimarker liquid biopsy panels for OC detection. At the same time, proteins associated with adhesion, extracellular matrix remodeling, immune suppression, oxidative stress adaptation and platinum resistance may provide information about the biological behavior of an individual tumor. Our bioinformatic analysis supports this concept, demonstrating that OC-associated exosomal proteins are involved in functionally related processes relevant to dissemination and treatment response. Therefore, exosomal proteome profiling may be valuable not only for the identification of general diagnostic markers, but also for stratifying patients according to metastatic and therapy-resistant tumor phenotypes. In the future, standardized EV isolation, quantitative proteomics and validation in independent clinical cohorts may allow exosomal protein signatures to be integrated into personalized therapeutic decision-making in OC.

## Figures and Tables

**Table 1:** Candidate EV-associated protein biomarkers reported for ovarian cancer liquid biopsy.

Candidate EV-Associated Protein(s)	Supporting OC EV Study Association	References
ACSL4, ITGA2, MYOF, FOLR1	Additional surface-accessible HGSOC EV candidates from the same lineage-informed study	[41]
AGRIN, CFH, PZP, CCNE1, FAS, SPP24, STAT3, PD-L1, IL6	Serum EV early-HGSOC panel	[66]
APOE, EpCAM, PLG, SERPINC1, C1Q	Plasma exosome diagnostic panel in epithelial OC	[25]
IGSF8, ITGA5, ITGB3	Lineage-informed HGSOC EV surface-marker study	[41]
ITGA6, ITGB2, ILK	Single-EV plasma classification panel	[42]
ITGB3/ITGB1/ITGA6 EV subpopulation	Tumor-associated single-EV subpopulation elevated in OC plasma	[42]
MUC1	Targeted plasma EV proteomics	[64]
MYL6, TTYH3, GSTP1	Early-stage HGSOC plasma sEV panel alongside MUC1	[65]
STX5, S100A4	Peritoneal-fluid sEV proteins associated with relapse/platinum-response biology	[50]

## Data Availability

The data presented in this study are available upon request from the corresponding author.

## Supplementary Materials

The following supporting information can be downloaded at: https://www.mdpi.com/article/10.3390/biom16081150/s1, Table S1: EV-associated protein entities included in the integrative analysis and supporting study characteristics; Table S2: Annotation coverage underlying Figure 3; Table S3: Complete GO Biological Process and Reactome enrichment statistics; Table S4: Pairwise Jaccard similarity of metabolic annotation profiles.

## Abbreviations

The following abbreviations are used in this manuscript:

BP	biological process
CT	computed tomography
EMT	epithelial–mesenchymal transition
EVs	extracellular vesicles
FIGO	International Federation of Gynecology and Obstetrics
GO	Gene Ontology
HE4	human epididymis protein 4
HGSOC	high-grade serous ovarian carcinoma
IOTA	International Ovarian Tumor Analysis
KEGG	Kyoto Encyclopedia of Genes and Genomes
OC	ovarian cancer
PPI	protein–protein interaction
ROMA	Risk of Ovarian Malignancy Algorithm
ROS	reactive oxygen species
sEVs	small extracellular vesicles
